# Determinants of seat belt use among drivers: a BRFSS study from a middle-income country (Vojvodina, Serbia)

**DOI:** 10.3389/fpubh.2026.1834226

**Published:** 2026-06-04

**Authors:** Smiljana Rajčević, Ivana Radić, Tanja Tomašević, Sonja Čanković, Sanja Harhaji, Snežana Ukropina, Katarina Mašić, Dragoslav Kukić, Mirjana Štrbac, Radmila Petrović, Tatjana Pustahija, Vladimir Petrović

**Affiliations:** 1 Institute of Public Health of Vojvodina, Novi Sad, Serbia; 2 University of Novi Sad, Faculty of Medicine, Novi Sad, Serbia; 3 University of Novi Sad, Faculty of Philosophy, Novi Sad, Serbia; 4 Department of Traffic Engineering, Faculty of Economics and Engineering, Novi Sad, Serbia; 5 University Clinical Center of Vojvodina, Novi Sad, Serbia

**Keywords:** behavioral risk factors, cross-sectional study, drivers, road safety, seat belt use

## Abstract

**Introduction:**

Wearing a seat belt is a crucial safety measure to prevent road traffic injuries, but adherence varies among different groups. Local data on driver behavior in Serbia, especially in the Autonomous Province of Vojvodina (APV), is limited.

**Objective:**

The aim of our study was to examine the prevalence of seat belt use and the sociodemographic factors influencing seat belt use among drivers in APV.

**Methods:**

A cross-sectional survey was conducted in 2023–2024 among 6,726 healthcare users aged ≥18 years. Participants were recruited from 44 primary healthcare centers across 45 municipalities using stratified sampling by age, sex, and settlement type. The questionnaire was adapted from the CDC Behavioral Risk Factor Surveillance System (BRFSS), and data were collected via Computer-Assisted Personal Interview (CAPI), using selected modules on demographics and seat belt use. The outcome was self-reported seat belt use (consistent/inconsistent). Independent variables included sociodemographic characteristics and BMI. Binary logistic regression was used to identify predictors of inconsistent seat belt use.

**Results:**

Overall, 83.1% of drivers reported always wearing a seat belt. Inconsistent use was significantly associated with male gender (AOR 0.54; 95% CI 0.47–0.63; *p* < 0.001), urban residence (AOR 1.34; 95% CI 1.16–1.55; *p* < 0.001), lower education (AOR = 0.44; 95% CI: 0.21–0.91; *p* < 0.027), self-employment (AOR 1.69, 95% CI 1.34–2.14, *p* < 0.001) or unemployment (AOR = 1.54; 95% CI: 1.11–2.13; *p* = 0.009), and certain districts, like Srem district (AOR = 1.94, 95% CI: 1.45–2.61; *p* < 0.001). Age, marital status, BMI, and self-assessed financial status were less consistent predictors.

**Conclusion:**

Seat belt use among APV drivers is high but varies by sociodemographic factors and region. Implementing targeted public health measures and local enforcement strategies is essential to improve compliance, especially among men, younger drivers, and high-risk occupational groups.

## Introduction

Road traffic injuries (RTIs) continue to be a significant public health issue worldwide, ranking among the leading causes of death and disability, especially among young and working-age adults. The World Health Organization (WHO) estimates that about 1.19 million people die each year in traffic crashes, with millions more suffering severe or lifelong injuries (1). RTIs create long-term physical, psychological, and social burdens for individuals and communities (2–5). Although there have been improvements in vehicle safety, traffic laws, and infrastructure management, significant disparities still exist between high-income and low- and middle-income countries, many of which still face high and mostly preventable rates of traffic injuries and deaths (6, 7).

Wearing a seat belt is one of the most effective ways to prevent road traffic injuries. Evidence shows that seat belts reduce the risk of fatal injuries by 45–50% and moderate to critical injuries by approximately 50% among drivers and front seat passengers (8, 9). Nevertheless, consistent seat belt use remains suboptimal worldwide (8, 10, 11). Compliance rates vary substantially across regions, with high levels reported in Europe, North America, and Australia, and lower, more heterogeneous rates in Asia, South America, and Africa (12, 13). In the European Union, front seat belt use exceeds 90%, while rear seat belt use particularly in Southern and Eastern Europe, remains markedly lower despite legal obligations (12). Increasing seat belt use is widely recognized as a cost-effective public health intervention and a key strategy for achieving the United Nations Decade of Action for Road Safety 2021–2030 target of reducing road traffic fatalities by 50% (13).

Seat belt use is influenced by a combination of individual, psychological, and contextual factors, including sociodemographic characteristics, risk perception, prior injury experience, and the effectiveness of law enforcement. Consistent evidence shows that men, younger individuals, and residents of rural areas are significantly less likely to wear seat belts regularly (8, 14). Differences are also observed between drivers and passengers, as well as between front and rear-seat occupants, highlighting the need for targeted, evidence-based interventions (15–17).

In Serbia, traffic law mandates seat belt use for both drivers and passengers; however, compliance remains inconsistent. National surveys show that while 86% of drivers and front-seat passengers wore seat belts in 2024, only 21% of rear-seat passengers did, with lower compliance among younger drivers and those in rural areas (18–21). Police reports indicate that 31% of victims in passenger vehicles were not wearing seat belts at the time of a crash, underscoring the ongoing public health issue (18). Regional differences reflect variations in cultural norms, access to information, and law enforcement effectiveness (22–24).

The Autonomous Province of Vojvodina (APV) offers a unique setting for examining behavioral risk factors for road safety in Central and Southeast Europe. Covering 21,614 km^2^ and home to nearly 2 million residents, Vojvodina features a dense road network, a mix of urban and rural communities, and high transit traffic (25, 26). These factors, along with its multicultural composition, shape driver attitudes and behaviors concerning road safety (21, 25).

Although many studies have examined seat belt use in high-income countries, evidence from Central and Southeast Europe remains limited, especially at the subnational level. Moreover, few studies have concurrently investigated sociodemographic and health-related factors that predict consistent seat belt use through standardized surveillance systems.

The study aimed to assess the prevalence of seat belt use among adults in Vojvodina. Additionally, the study aimed to explore the relationships among sociodemographic factors, BMI, and seat belt use among adults in APV, Serbia, using data from a newly established Behavioral Risk Factors Surveillance System.

## Materials and methods

### Study design and population

The study was conducted as a cross-sectional survey. Data collection was part of the larger “Program Task of the Special Public Health Program for the Territory of APV: Surveillance of Behavioral Risk Factors for Non-Communicable Diseases in the Adult Population of APV (SBRF-NCD-V),” implemented by the Institute of Public Health of Vojvodina (IPHV) in accordance with government regulations (“Official Gazette of APV,” Nos. 54/2022 and 45/2023) (27, 28).

The target population included adults aged ≥18 years who were registered users of Primary Healthcare Centers (PHCs) at all 44 PHCs across in all 45 municipalities of Vojvodina Participants were recruited during routine visits to PHCs, where they accessed services such as general medicine, occupational health, gynecology, child healthcare (parents accompanying children), and polyvalent services, ensuring broad population coverage and a sample representative of the province’s adult population. Within PHCs, trained interviewers recruited participants consecutively, following the planned stratification quotas to ensure that the final sample reflected the population structure.

Eligible participants were adults aged 18 years or older who were registered users of PHCs in the APV. Participants needed to understand the survey questions and give informed consent. Individuals were excluded if they were under 18 years old, not registered users of PHCs in Vojvodina. Additionally, those with severe mental impairments that could affect their ability to provide reliable responses, as well as anyone who refused to participate in the study, were excluded from the analysis.

For this study, analyses focused only on respondents who identified as drivers. Seat belt use was evaluated solely among these individuals. Those who reported never driving, as well as those who refused to answer or were unsure of their driving status, were excluded from the analysis.

No sample weights were used; however, representativeness was ensured through stratified sampling by age, sex, and settlement type, with proportional allocation across districts and municipalities based on census data data.

Two separate survey waves took place in 2023 and 2024, using the same protocols, sampling methods, and data collection procedures. This strategy enabled combining the data to improve statistical power and accuracy while accounting for survey year as a covariate in statistical analyses.

### Sampling and sample size

A representative sample of adults was selected using a stratified, two-stage sampling design that considered gender, age group, settlement type (urban/rural), and district. Sampling proportions were based on the latest census data (29), with proportional distribution across the seven districts and 45 municipalities of Vojvodina. The target sample size for each wave was determined using a smoking prevalence of 35.5% (a key behavioral risk factor), with a 1.5% margin of error and 95% confidence level. The two survey waves combined yielded 7,820 respondents (3,910 per year) for analysis.

### Data collection

Data were collected from May to August during each survey year. The questionnaire was based on the CDC’s (Centers for Disease Control and Prevention, United States) BRFSS (Behavioral Risk Factor Surveillance System) questionnaire. Creating the questionnaire involved selecting questions from the BRFSS across different years (for the purpose of this paper, 2020) (30). The following BRFSS modules were used: Demographics and Seat Belt Use. Questions from the modules Demographics and Seat Belt Use were translated into Serbian and culturally adapted. No validation of the questionnaire was done, since the BRFSS is an internationally validated instrument widely used in population health surveillance. A pilot study was carried out in 2023 with a sample of 30 respondents.

Computer-Assisted Personal Interviews (CAPI) were conducted face-to-face by trained health professionals using mobile devices with secure, authorized access to survey software. Forty-five field teams were organized to collect data across all municipalities. Interviewer training included detailed guidelines for fieldwork, supervision, and quality control, such as automated validation checks and skip patterns to ensure data completeness and consistency.

### Variables

Dependent variable: seat belt use was measured by asking participants how often they wear seat belts while driving, with response options categorized as always” versus all other frequencies (“almost always,” “sometimes,” “rarely,” “never”) for logistic regression analyses. As this study focuses on seat belt use while driving, all analyses were restricted to respondents who reported driving a car, although the original survey targeted the general adult population.

Independent variables: Sociodemographic and health-related factors included age, gender, settlement type, district, marital status, level of education, self-assessed financial situation, employment status, and body mass index (BMI). The survey year was also included as an independent variable to account for potential changes over time between waves.

### Ethical considerations

The study protocol, questionnaire, and informed consent procedures were reviewed and approved by the Ethics Committee of the Institute of Public Health of Vojvodina (Decision No. 01–745/2, April 18, 2023). Participation was voluntary, and all participants provided written informed consent before enrolling.

### Statistical analysis

Data were analyzed using SPSS version 26. Descriptive statistics included frequency distributions and percentages for categorical variables, and mean ± standard deviation for continuous variables. Differences between groups were assessed using the Chi-square (*χ*^2^) test for categorical variables.

Because of the identical study design, sampling strategy, and data collection procedures, data from the 2023 and 2024 survey waves were pooled to enhance the statistical power and accuracy of the estimates. The survey year was included as an independent variable in statistical analyses to address possible temporal differences.

Multivariable analysis was performed using binary logistic regression to identify factors independently associated with seat belt use. Adjusted Odds Ratios (AORs) with 95% confidence intervals (CIs) were provided for all predictors. Multicollinearity was checked using the Variance Inflation Factor (VIF) and Tolerance values (VIF range: 1.01–1.54; Tolerance range: 0.65–0.99), confirming there was no significant collinearity among independent variables. Cases with missing or invalid responses (e.g., “do not know” or “prefer not to answer”) were excluded. All independent variables included in the regression models were categorical with clearly defined reference categories. Statistical significance was set at *p* ≤ 0.05.

## Results

A total of 7,820 participants completed both survey waves. Respondents who never drove a car (*n* = 1,083), those who refused to answer (*n* = 7), or were unsure (*n* = 4) were excluded. As a result, the final number of respondents in the analysis is 6,726 (2023: 2,981, 2024: 3,745). The average age was 47.7 years (SD = 17.5; range 18–93), and 51.7% were male. The most common age group was 55 years or older (36.9%). Most participants had completed secondary education (59.3%), were employed (58.4%), and lived in urban areas (60.5%). Sociodemographic characteristics of the study population are presented in Table 1.

**Table 1:** Sociodemographic characteristics of the study population, by seat belt use, among adult drivers in Vojvodina.

Sociodemographic characteristics	Seat belt use				
	Always		Inconsistent use		*p*
	*n*	%	*n*	%	
Age (years)					
18–24	512	81.4	117	18.6	0.325
25–34	930	83.5	184	16.5	
35–44	1,092	83.0	224	17.0	
45–54	1,004	84.9	179	15.1	
55+	2049	82.5	434	17.5	
Gender					
Male	2,747	79.0	731	21.0	<0.001
Female	2,841	87.5	407	12.5	
Settlement place					
Urban	3,483	85.6	588	14.4	<0.001
Other	2,105	79.3	550	20.7	
District					
North Bačka	576	87.3	84	12.7	<0.001
Central Banat	512	81.3	118	18.7	
North Banat	395	81.3	91	18.7	
South Banat	791	84.3	147	15.7	
West Bačka	526	81.3	121	18.7	
South Bačka	1968	85.5	335	14.5	
Srem	820	77.2	242	22.8	
Marital status					
Married	3,257	83.6	638	16.4	0.044
Divorced	438	80.7	105	19.3	
Widow/widower	455	82.9	94	17.1	
Separated	29	67.4	14	32.6	
Never married	1,131	83.4	225	16.6	
Living with a partner	251	81.2	58	18.8	
Education level					
Incomplete primary school/no school	39	67.2	19	32.8	<0.001
Primary school	452	78.3	125	21.7	
Secondary school	3,268	82.0	717	18.0	
University degree	1822	86.9	274	13.1	
Self-assessed financial situation					
Very good	429	84.4	79	15.6	0.003
Good	2095	83.4	416	16.6	
Average	2,628	83.7	513	16.3	
Poor	350	77.3	103	22.7	
Very poor	45	73.8	16	26.2	
Employment status					
Employed	3,303	84.3	614	15.7	<0.001
Self-employed	359	73.0	133	27.0	
Unemployed 1 year or more	199	72.9	74	27.1	
Unemployed for less than 1 year	115	79.3	30	20.7	
Housewife	180	82.6	38	17.4	
Student	243	84.7	44	15.3	
Retired	1,125	85.3	194	14.7	
Unable to work	54	85.7	9	14.3	
BMI					
BMI < 18.5	98	90.7	10	9.3	0.002
BMI 18.5–24.9	2,306	85.2	399	14.8	
BMI 25.0–29.9	1987	81.9	439	18.1	
BMI ≥ 30	926	82.6	195	17.4	
Total	5,588	83.1	1,138	16.9	

**Figure 1:**
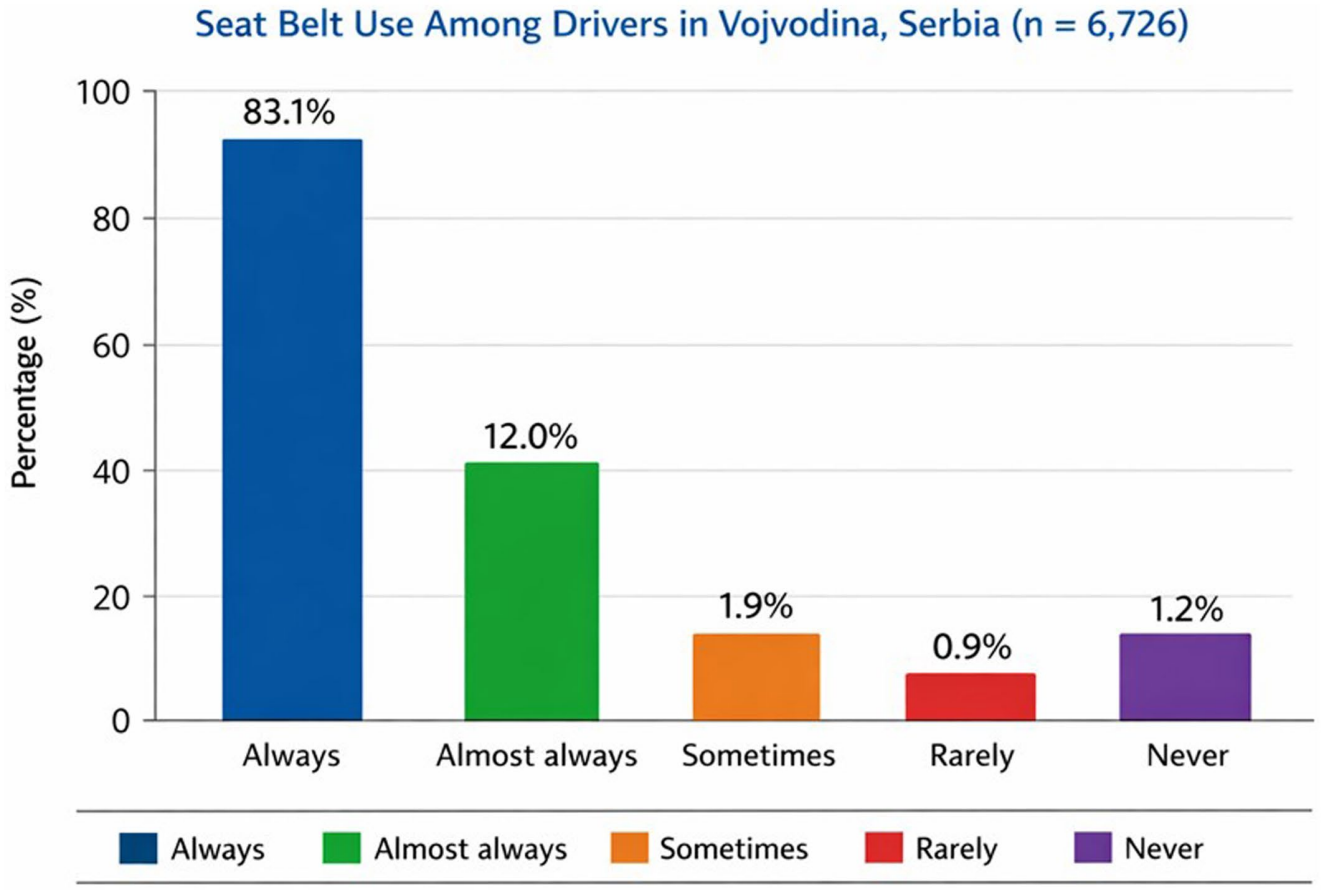
The prevalence (%) of seat belt use among adult drivers in Vojvodina, Serbia.

Overall, 83.1% of participants reported consistently wearing a seat belt while driving, while 16.9% reported inconsistent use (“almost always,” “sometimes,” “rarely,” or “never”) (Figure 1). Univariable analyses using Chi-square tests showed significant associations between seat belt use and sex, type of settlement, district, education level, employment status, BMI, and self-assessed financial situation (all *p* < 0.05). (Figure 1).

The assumptions of the binary logistic regression model were statistically significant (*χ*^2^ (35) = 273.81, *p* < 0.001) and correctly classified 83.6% of cases, explaining 7.2% of the variance in seat belt use.

Multivariable binary logistic regression analysis was conducted to identify independent predictors of inconsistent seat belt use (Table 2). Female drivers had significantly lower odds of inconsistent seat belt use compared to males (AOR 0.54, 95% CI 0.47–0.63, *p* < 0.001). Drivers aged 45–54 were less likely to report inconsistent use compared to the youngest age group (AOR 0.67, 95% CI 0.47–0.97, *p* = 0.034). Urban residence (AOR 1.34, 95% CI 1.16–1.55, *p* < 0.001) and living in Srem (AOR 1.94, 95% CI 1.45–2.61, *p* < 0.001), Central Banat (AOR 1.69, 95% CI 1.22–2.34, *p* = 0.002), or North Banat (AOR 1.58, 95% CI 1.12–2.23, *p* = 0.009) districts were linked to increased odds of inconsistent use. Education demonstrated a transparent gradient: participants with higher levels of education were more likely to wear seat belts consistently (University degree: AOR 0.27, 95% CI 0.13–0.58, *p* < 0.001). Employment status was also a significant factor, with self-employed (AOR 1.69, 95% CI 1.34–2.14, *p* < 0.001) and long-term unemployed drivers (AOR 1.54, 95% CI 1.11–2.13, *p* = 0.009) showing higher odds of inconsistent use, while retirees were more compliant (AOR 0.68, 95% CI 0.53–0.88, *p* = 0.003). BMI and self-rated financial status were not significant predictors in the multivariable model. Lastly, participants surveyed in 2024 were more likely to report inconsistent use than those surveyed in 2023 (AOR 1.26, 95% CI 1.09–1.45, *p* = 0.001).

**Table 2:** Multivariable binary logistic regression analysis of factors associated with inconsistent seat belt use among adult drivers in Vojvodina.

Predictor	Category	AOR	95% CI	*p*
Age	18–24	Ref.		
	25–34	0.78	0.56–1.08	0.134
	35–44	0.77	0.54–1.09	0.142
	45–54	0.67	0.47–0.97	**0.034**
	55+	0.85	0.59–1.22	0.370
Gender	Male	Ref.		
	Female	0.54	0.47–0.63	**0.000**
Settlement place	Other	Ref.		
	Urban	1.34	1.16–1.55	**0.000**
District	North Backa	Ref.		
	Central Banat	1.69	1.22–2.34	**0.002**
	North Banat	1.58	1.12–2.23	**0.009**
	South Banat	1.16	0.85–1.58	0.345
	West Backa	1.53	1.10–2.13	0.012
	South Backa	1.20	0.91–1.59	0.185
	Srem	1.94	1.45–2.61	**0.000**
Marital status	Married	Ref.		
	Divorced	1.26	0.94–1.57	0.132
	Widow/widower	1.28	0.95–1.72	0.112
	Separated	2.51	1.24–5.08	**0.011**
	Never married	0.96	0.76–1.21	0.733
	Living with a partner	1.26	0.91–1.74	0.157
Education level	Incomplete primary school/no school	Ref.		
	Primary school	0.44	0.21–0.91	**0.027**
	Secondary school	0.36	0.18–0.74	**0.005**
	University degree	0.27	0.13–0.58	**0.000**
Self-assessed financial situation	Very good	Ref.		
	Good	1.10	0.83–1.46	0.500
	Average	0.96	0.75–1.32	0.974
	Poor	1.32	0.91–1.91	0.136
	Very poor	1.407	0.69–2.87	0.349
Employment status	Employed	Ref.		
	Self-employed	1.69	1.34–2.14	**0.000**
	Unemployed 1 year or more	1.54	1.11–2.13	0.009
	Unemployed for less than 1 year	1.21	0.77–1.89	0.409
	Housewife	1.03	0.68–1.55	0.898
	Student	0.79	0.51–1.23	0.299
	Retired	0.68	0.53–0.88	**0.003**
	Unable to work	0.51	0.21–1.23	0.135
BMI	BMI < 18.5	Ref.		
	BMI 18.5–24.9	1.34	0.68–2.63	0.394
	BMI 25.0–29.9	1.47	0.74–2.89	0.270
	BMI ≥ 30	1.45	0.72–2.88	0.295
A year of research	2023	Ref.		
	2024	1.26	1.09–1.45	**0.001**

95% CI, 95% confidence interval; Ref, reference value; AOR, adjusted odds ratio; in bold are presented statistically significant p values (*p* < 0.05).

## Discussion

This study provides population-based evidence on seat belt use among drivers in the Vojvodina, using data from the recently established Surveillance System of Behavioral Risk Factors for Non-Communicable Diseases (SBRF-NCD-V). The findings show that seat belt use is influenced by a mix of individual, socioeconomic, and regional factors, reflecting broader social inequalities in preventive health behavior.

The overall prevalence of consistent seat belt use (83%), was slightly lower than recent national estimates from Serbia (86%) (18) and similar to levelsreported in some neighboring countries, such as Croatia (92%) front-seat use in 2019 (31). Across the Western Balkans, Serbia generally shows higher compliance, while most other countries report lower rates, often around 30%–40% (32). Globally, compliance varies widely, with the highest rates in high-income countries with long-standing enforcement traditions like Germany, Canada, and Japan, and much lower levels in several low- and middle-income settings such as parts of Asia, the Middle East, and Africa (14, 33, 34). The present findings indicate that although compliance in Vojvodina is relatively high, significant gaps still exist within certain population subgroups. These differences mainly result from variations in legislation, law enforcement, safety culture, and public awareness, underscoring the importance of ongoing prevention efforts, enhanced police oversight, and targeted local interventions.

Our multivariable analysis identified several key sociodemographic predictors of inconsistent seat belt use. Gender proved to be a strong and consistent predictor of seat belt use. Male drivers were notably more likely than female drivers to report inconsistent use, a pattern observed across Europe and worldwide. The ongoing presence of this pattern in Vojvodina suggests that behavioral interventions targeting male drivers should remain a priority (7, 35).

Age differences were less noticeable. Although younger drivers (17–23) showed slightly lower compliance, only the 45–54 age group had significantly higher odds of consistent seat belt use compared to the youngest group. This partly aligns with previous evidence suggesting that middle-aged and older drivers tend to adopt more cautious driving behaviors. However, the lack of a clear linear age trend indicates that seat belt use in this population may be more strongly affected by socioeconomic and contextual factors than by age alone (36–39). Nevertheless, these findings emphasize the ongoing importance of early traffic safety education and targeted interventions for young drivers.

The finding that urban residents are more likely to report inconsistent seat belt use than those living in other settlements deserves particular attention. This contrasts with the common belief that urban areas—thanks to better infrastructure and higher enforcement visibility—would show greater compliance. One possible explanation is a “familiarity effect,” in which routine short-distance driving in perceived low-risk environments reduces the perceived need for consistent seat belt use. Additionally, higher traffic density, frequent stops, and lower average speeds may also reduce the perceived severity of crashes (40–42). This finding aligns with evidence from spatial epidemiological studies showing that seat belt use is not randomly distributed but instead forms geographically structured clusters influenced by local socioeconomic and contextual factors (38, 43). Hezaveh et al. demonstrated significant spatial autocorrelation in seat belt non-use, indicating that behavioral patterns tend to cluster across neighboring geographic areas rather than being randomly distributed. Their findings also suggest that urban density, population structure, and mobility patterns in the local context contribute to spatial differences in compliance beyond individual-level factors (44). Regional disparities within Vojvodina were also evident, with some districts showing significantly lower compliance compared to North Bačka. Similar intra-regional differences have been observed in European countries and are often linked to variations in enforcement levels, local safety culture, and socioeconomic makeup (14, 45–47). These findings emphasize that traffic safety behaviors are influenced not only by personal characteristics but also by local environmental factors. Therefore, interventions should be geographically tailored rather than uniformly implemented across the entire province.

One of the most notable findings relates to the strong educational gradient. Higher educational attainment was independently associated with significantly lower odds of inconsistent seat belt use, with university-educated drivers showing much higher compliance. This gradient indicates that seat belt use reflects broader social determinants of health, such as access to information, health literacy, risk perception, and adherence to safety norms. These results align with previous studies that connect higher education and socioeconomic status to improved safety behaviors (10, 48–50). The findings, therefore, emphasize that improving compliance might require structural and educational approaches rather than relying solely on enforcement. Employment status further reinforced this social pattern. Self-employed and long-term unemployed individuals had higher odds of inconsistent seat belt use, while retirees showed better compliance. These relationships may reflect differences in daily mobility routines, perceived time constraints, occupational risk perception, and overall socioeconomic vulnerability. Alongside the educational gradient, these findings suggest that safe driving habits are rooted in broader social and economic factors.

The observed increase in inconsistent seat belt use in 2024 compared to 2023 is notable. Although the two survey waves were sampled independently, the time gap may represent changes in enforcement practices, public awareness efforts, or evolving attitudes toward traffic laws. Continuous monitoring is vital to determine if this is a short-term fluctuation or an emerging trend.

Although BMI and self-assessed financial situation were not significant predictors in the analyses, descriptive trends indicated lower compliance among overweight or socioeconomically disadvantaged individuals (51, 52). These patterns may reflect ergonomic discomfort, attitudinal differences, or broader associations between lower socioeconomic status and weaker adherence to preventive health behaviors (53, 54).

Overall, the findings show that seat belt use in Vojvodina is influenced by ongoing sociodemographic inequalities, especially educational level, employment status, and regional factors. The observed social gradient indicates that safe driving behavior is shaped by broader structural determinants rather than solely by personal choice. Although overall compliance was fairly high, certain groups—especially men, self-employed and long-term unemployed individuals, and residents of specific districts—remain at higher risk of inconsistent seat belt use.

These findings align with earlier research emphasizing the impact of socioeconomic and contextual factors on seat belt compliance (7, 14). For example, a recent spatiotemporal analysis of telematics data from 99 counties in Iowa demonstrated that vehicle miles traveled and per capita income were key predictors of seat belt use (45). This highlights the importance of spatial and socioeconomic elements in understanding safety behaviors. The study also emphasized the usefulness of high-resolution telematics data for detecting geographic and temporal patterns and guiding targeted traffic safety initiatives. This evidence suggests that broader contextual and structural factors influence seat belt use, beyond just individual characteristics. Overall, these recent findings indicate that seat belt use in Vojvodina reflects wider structural and behavioral influences consistent with trends seen across Europe.

Unlike high-income regions where technology and compliance are widespread, countries in Central and Eastern Europe, including Romania and Serbia, still show differences in seat belt use, especially among younger men (55). Recent evidence from Romania indicates that although overall seat belt use has increased in recent years, persistent disparities remain by gender, age, and education, with lower compliance consistently observed among younger male drivers and individuals with lower educational attainment (56). These findings align with our results and suggest that socioeconomic gradients in preventive road safety behavior are stable across similar transitional settings.

Public health strategies should therefore combine strong law enforcement with targeted, equity-focused interventions. Customized educational campaigns, especially for socially vulnerable groups and high-risk districts, along with consistent police oversight and culturally adapted communication methods, can help reduce disparities and further improve compliance. Ongoing monitoring of behavioral risk factors is vital for tracking trends and assessing the effectiveness of interventions over time.

## Limitations

This study has several limitations. First, seat belt use was self-reported and may be affected by recall and social desirability biases, potentially leading to an overestimation of compliance. Second, although the sample was representative of the Autonomous Province of Vojvodina, estimates for smaller subgroups and certain districts might be less accurate due to limited sample sizes. Third, socioeconomic status was assessed based on self-perceived financial situation rather than objective income data, which may not fully reflect material conditions. In addition, BMI was not directly measured but calculated from self-reported body weight and height, which may introduce reporting bias and misclassification. Sampling weights were not used in the analysis, which may affect the precision of population-level estimates. Although two survey waves were analyzed, the samples were selected independently; therefore, conclusions about changes over time at the individual level cannot be drawn. Lastly, the regression model explained only a small portion of the variation in seat belt use, suggesting that other psychosocial, behavioral, and contextual factors not included in this survey may also be important. Despite these limitations, the study provides valuable population-level evidence on sociodemographic and regional patterns of seat belt use in Vojvodina, improving understanding of behavioral risk factors in traffic safety.

## Conclusion

Seat belt use among adult drivers in Vojvodina is quite high, with over 80% reporting consistent use. However, nearly one in six drivers admits to inconsistent use, posing a significant preventable risk for traffic-related injuries and deaths. The findings show that seat belt use is influenced by several sociodemographic factors. Male drivers, younger individuals, urban residents, those with lower education levels, self-employed and long-term unemployed people, and residents of the Srem, Central Banat, and North Banat districts were identified as groups at higher risk of not consistently using seat belts. Additionally, the rise in inconsistent seat belt use in 2024 compared to 2023 highlights a concerning temporal trend that needs further observation. These findings underscore the importance of targeted, evidence-based public health efforts, especially educational campaigns aimed at high-risk groups, such as men, younger drivers, and those with lower levels of education.

The regional disparities identified in this study highlight the need for locally tailored interventions in districts with lower compliance rates. Enhancing enforcement of seat belt laws, especially in urban areas, could further boost adherence. Workplace health promotion efforts may also be helpful, particularly for self-employed and unemployed groups. Additionally, ongoing behavioral monitoring systems are vital for tracking trends, spotting new risk groups, and assessing the success of interventions. Collaboration among public health agencies, traffic safety authorities, policymakers, and local communities is essential to developing comprehensive, sustainable strategies to increase seat belt use. Overall, these findings offer valuable evidence to guide targeted traffic safety policies and public health efforts to reduce preventable injuries and deaths in Vojvodina and similar regions.

## Data Availability

The raw data supporting the conclusions of this article will be made available by the authors, without undue reservation.
